# Incorporating digital health into organizational health literacy: An updated definition, tools, and recommendations

**DOI:** 10.1177/08404704251356518

**Published:** 2025-07-08

**Authors:** Helen Monkman, Blake J. Lesselroth

**Affiliations:** 1 8205 University of Victoria, Victoria, British Columbia, Canada.; 2 161413 University of Oklahoma, Tulsa, Oklahoma, United States of America.

## Abstract

Health literacy is important from two perspectives: the individuals (personal health literacy) and the organizations providing information and services (organizational health literacy). While research has addressed digitalization in healthcare and associated barriers and enablers in personal health literacy (e.g., digital health literacy), these developments have not been paraleled in organizational health literacy. In this article, we proposed an augmented definition of organizational health literacy and conducted a gap analysis of the Health Literacy Universal Precautions Toolkit to expand it for digital health. Important advancements, specifically for virtual care, have been made, yet a broader approach must be adopted for all digital health technology. We proposed a series of modifications to emphasize the importance of digital health in organizational health literacy. Organizations must equitably enable individuals to understand and use digital information and services. In this monograph, we describe the current informatics gap and the required competencies, policies, and infrastructure to close the gap.

## Introduction

Organizations and researchers have defined and interpreted health literacy differently over time.^1,2^ Nearly all early definitions (see Parnell^1^ example) emphasize individual skills and abilities. However, it is increasingly apparent that the healthcare system and stakeholders mediate health literacy.^3^ That is, healthcare is interactive: individuals seeking and receiving care—and healthcare professionals interacting with systems—collectively influence communication success. Consequently, in 2020, the Center for Disease Control (CDC) updated its health literacy definition by introducing personal and organizational constructs.^4^ Personal Health Literacy (PHL) emphasizes individual agency. It is defined as “the degree to which individuals have the ability to find, understand, and use information and services to inform health-related decisions and actions for themselves and others.”^4^ Citizens need PHL to perform health system navigation and engagement tasks (e.g., booking appointments, articulating symptoms, and following treatment plans).^5^ In contrast, Organizational Health Literacy (OHL) captures the role and responsibility of the healthcare system. It is “the degree to which organizations equitably enable individuals to find, understand, and use information and services to inform health-related decisions and actions for themselves and others.”^4^

Health literacy universal precautions are a systematic approach to patient engagement that assumes everyone may struggle finding, accessing, understanding, and using health information and services, irrespective of socioeconomic status, education, language, and literacy.^6,7^ Practitioners applying universal communication precautions provide “health information and services in ways that everyone can understand and use.”^8^ Examples to improve OHL through universal precautions include “simplifying the process to schedule appointments, using the Teach-Back method to ensure patient comprehension, and providing communications in the appropriate language, reading level, and format.”^9^ While organizations are making progress in OHL,^10^ introduction of digital information and communication technologies creates new service delivery challenges. Thus, we must update our definitions and expectations for a digital context.

### An updated definition of organizational health literacy

Health Literacy (HL) researchers have identified new constructs to capture the *individual* skills necessary for successful interactions with the healthcare system and health information in the context of the internet (i.e., eHealth literacy^11^). Additional skills are necessary given a growing digital ecosystem and the emergence of medical tools such as patient portals, mobile applications, and wearable sensors.^12,13^ These advancements motivated the need to conceptualize Digital Health Literacy (DHL), a term first introduced in 2012.^14^ Although eHealth literacy is often used interchangeably with DHL, the latter is more comprehensive. Some consider digital health literacy a super social determinant of health.^15,16^ We believe DHL is context-dependent, encapsulating skills that vary as a function of the device, operating system, application, health topic, or type of information.

Technology can facilitate or impede health literacy^13^ and thus has the potential to narrow or widen disparities.^17^ For example, artificial intelligence and machine learning, voice commands and speech recognition, wearables, remote monitoring, and mobile applications can all help build individuals’ DHL. Conversely, introducing any of these technologies, indiscriminantly and without addressing other social determinants of health, including technology access, infrastructure (e.g., high-speed broadband internet), and usability, can disenfranchise users. Therefore, we must ensure that technology demands do not exceed users’ skills and capabilities.^18^ In summary, while content (i.e., HL) is critically important, we must also consider the delivery medium (i.e., DHL) and the role OHL plays to responsibly and effectively deploy digital health initiatives.

The World Health Organization’s Global Strategy on Digital Health 2020-2025^19^ asserts that

Digital health will be valued and adopted if it: is accessible and supports equitable and universal access to quality health services; enhances the efficiency and sustainability of health systems in delivering quality, affordable, and equitable care; and strengthens and scales up health promotion, disease prevention, diagnosis, management, rehabilitation, and palliative care including before, during, and after an epidemic or pandemic, in a system that respects the privacy and security of patient health information.

Given that the personal health literacy has evolved to reflect the increasing reliance on information communications and technologies by introducing constructs such as eHealth and DHL, OHL should naturally be updated as well. We propose a simple yet important modification to the definition of OHL to address emerging organizational challenges: adding digital and analogue. We propose that OHL is the degree to which organizations equitably enable individuals to find, understand, and use *digital and analogue* information and services to inform health-related decisions and actions for themselves and others.

### Objectives

This augmented definition motivates a need to expand health literacy universal precautions to address the complexities of digital healthcare. We take a pragmatic approach by examining existing tools supporting OHL and providing considerations for a holistic perspective of OHL that includes the complexities of digital health. Thus, the objectives of this article are to (1) identify where tools in Health Literacy Universal Precautions Toolkit (3^rd^ edition)^7^ currently consider the digital health context, (2) highlight opportunities to expand existing tools for the digital health context, and (3) provide recommendations for future tool development.

## The Health Literacy Universal Precautions Toolkit

To help improve OHL, the Agency for Healthcare Research and Quality (AHRQ) published a Health Literacy Universal Precautions Toolkit, currently in its 3rd edition.^7^ Universal precautions for health literacy emphasize:

• “Simplifying communication and confirming understanding with everyone,
• Making offices and the healthcare system easier to navigate, and
• Supporting people’s efforts to improve their health.”^8^

The Toolkit contains 23 tools with evidence-based actions organized into five sections:

(1) Tools to start on the path to improvement,
(2) Tools to improve spoken communication,
(3) Tools to improve written communication,
(4) Tools to improve self-management and empowerment, and
(5) Tools to improve supportive systems.^8^

## Existing and proposed considerations for digital health in tools from the Health Literacy Universal Precautions Toolkit

We reviewed all 23 tools in the Health Literacy Universal Precautions Toolkit (3^rd^ edition)^7^ for any references to digital technology (e.g., Electronic Health Records (EHRs), patient portals, and remote patient monitoring). We documented existing recommendations and proposed new considerations to address the digital health landscape (see Table 1).

**Table 1.** Existing considerations and recommended complements to the health literacy universal precautions toolkit to for the digital context.

Section	Health literacy universal precautions tool	Existing considerations for the digital context	Recommended complement for the digital context
1) Tools to start on the path to improvement			
	Form a team: Tool #1		The team leader should champion and facilitate expanding adoption of digital health technologies
	Assess organizational health literacy and create an improvement plan: Tool #2		Include assessment of digital health
	Raise awareness: Tool #3		Raise awareness about digital health literacy, digital health equity, and the facilitators and barriers to adopting digital health technologies
2) Tools to improve spoken communication			
	Communicate clearly: Tool #4		Use digital tools to assist with language translation
			Provide written and multimedia materials to patients to enrich understanding and facilitate recall (e.g., exercise, taking medications, and chronic illness monitoring)
	Use the teach-back method: Tool #5		
	Follow-up with patients: Tool #6	Already includes asking about contact preferences and providing options (e.g., secure e-mail and texting,) and using EHRs or digital calendars for tracking follow-up	
		Already includes recommending digital technologies that may make automatically transfer data	
	Be easy to reach: Tool #7	Already includes making e-mail systems patient-friendly and selecting easy to use patient portals	Potentially need to add conversational agents or chatbots to help patients reach out and connect with organizations
	Conduct brown bag medicine reviews: Tool #8		Telemedicine and remote monitoring create more opportunities for brown bag reviews
			Consult other existing medication lists (e.g., health information exchange and provincial pharmacy networks) for compared with brown bag reviews
			Consider digital tools specifically to support medication reconciliation^20,21^
	Address language differences: Tool #9	Already includes providing written materials and videos in patients’ languages (including sign language) and considering options for multilingual patient portals	Telemedicine increases the availability and breadth of qualified interpreters. Use closed captioning
	Consider culture: Tool #10	Already includes web sites and courses for opportunities to learn	Include Canadian specific references
3) Tools to improve written communication			
	Assess, select, and create easy-to-understand materials: Tool #11	Already includes using the Health Literacy Online: A Guide for Simplifying the User Experience^22^ to support the design and revision of web sites	Refer readers to specific advice for mobile devices^23^ in Health Literacy Online^22^
			Refer readers directly to the Health Literacy Online Strategies Checklist^24^ to support assessment
			Use the (SHeLL) Sydney Health Literacy Lab Health Literacy Editor (available here: https://shell.techlab.works/)
	Use health education material effectively: Tool #12	Already mentions that simply referring patients to a web site is inadequate and does not ensure understanding or behaviour change	Adapt recommendations for patient portals to other devices, apps, web sites, and tools Include patient education about high-quality resources, the limits of generative artificial intelligence, and the risks of emerging technologies
		Already mentions it is necessary to ensure patients know how to use the videos and audiovisual materials or access the internet. Refers to recommendations for getting patients set up with telehealth technology^25^	
		Already includes “Train patients to use the patient portal and to be discerning consumers of internet content.” Already recommends observing patients using the portal and collecting feedback using the Patient Portal Feedback Form.^26^ Most questions are binary with open-ended feedback to improve suboptimal tasks^26^	
	Welcome patients: Tool #13	Already suggests offering to assist patients with completing forms, using patient portals, and telehealth	Ensure organization’s web site includes all the information recommended for its brochure (e.g., contact information, services provided, address, and map)
			Consider digital maps and wayfinding technology to support patients navigating healthcare environments and procedures
			Consider kiosks or loaned devices to help patients check in for appointments and collect intake data
			Consider using a digicaster to showcase information in the waiting room
4) Tools to improve self-management and empowerment			
	Encourage questions: Tool #14	Already recommends using Question Builder^27^ online or app to prepare questions for appointments. Question Builder generates relevant questions based on the patient’s response to appointment type.^27^ Patients can print their questions to bring with them to their appointment and write in the responses^27^	Include options to generate questions or discussion guides for patients using personal health portals, apps, or e-mails
			Explore role of conversational agents or chatbots Questions could be generated based on the patient’s context (e.g., age, sex, diagnoses, treatment, and medication). Artificial intelligence may play a role in providing high-quality health information, developing treatment plans, recommending resources, offering plant-based recipes and graduated exercise prescriptions, or generating patient-friendly medical instructions
	Make action plans: Tool #15		Consider including options for apps and devices to support specific interventions and behaviour change
	Help patients take medicine correctly: Tool #16	Already recommends incorporating evidence- based, explicit, and standardized prescription medication instructions^28^ into EHRs for e-prescribing	Determine if more comprehensive lists of medications are available (e.g., health information exchanges and provincial pharmacy networks) and if they can be augmented to include more details to make them patient-centric and include other items (e.g., vitamins and over the counter medications) Although costly, automated medication dispensers may also be considered
		Already recommends using EHRs, where possible, to generate patient-centred medication lists that include additional details (e.g., what the medication is for, and times of day it should be taken) Structured Word and PDF forms also recommended as alternatives but require manual data entry	
		Already recommends helping choose and set up electronic reminders, apps, smart bottles, and pill boxes	
	Get patient feedback: Tool #17	Already recommends observing patients using the patient portal to identify opportunities for improvement, collecting feedback using the Patient Portal Feedback Form,^26^ and providing this information to portal vendors	Deploy regular feedback loops and collect remote usability metrics to monitor quality, improve products, and ensure organizational accountability Need to add survey questions to specific to other types of technology
		Already provides survey questions for different tools in the Toolkit^29^	
		Already includes option of administering web-based surveys with the limitation that they likely will not garner feedback from people who use technology less	
		Already recommends including an audio option. Already warns against using patient portals for surveys to minimize bias of who is more likely to use portals	
5) Tools to improve supportive systems			
	Attend to social needs: Tool #18	Already recommends determining if EHR has social screening questions embedded, documenting social needs in the EHR using ICD-10-CM Z Codes for Social Determinants of Health, and using the EHR for support referrals	Canadian specific information must be included and insertion points for other federal or provincial health authorities
	Help patients pay less for medicine: Tool #19		Many of these recommendations do not apply in Canada
	Connect patients with literacy and math resources: Tool #20	Already recommends referring patients to local resources using the EHR	Add digital literacy resources. For example, Connected Canadians offers free technology training and support for older adults^30^
	Make referrals easy: Tool #21	Already recommends using electronic referrals through the EHR or a stand-alone system	Some of the cost information may not be relevant in the Canadian context May want to consider sharing the status of referrals and appointment preparation instructions digitally through secure messaging
	Include family and friends: Tool #22	Already includes recommendation for including a statement encouraging patients to bring family or friends if appointments are booked online	Needs recommendation for private time with patient in telehealth appointments
		Already recommends having support person and patient in camera view during telehealth appointments	
	Talk about costs: Tool #23		This is mostly irrelevant in the Canadian context

### Tools to start on the path to improvement

This section did not mention technology. We recommend identifying a team leader or clinical champion responsible for expanding offerings and promoting the adoption of digital health technologies. Organizations should analyze their strengths and weaknesses supporting digital health offerings and implement educational initiatives for staff about DHL, digital health equity, and barriers and facilitators to adopting digital health technology.

### Tools to improve spoken communication

The Toolkit encourages professionals to ask patients about their communication preferences and offer digital and analogue options. It also recommends using EHRs or digital calendars to track patient follow-up. When home monitoring is necessary, it is crucial to align patient preferences for reporting and data collection (e.g., patient portals, apps, and devices that automatically transmit data). Citizens should be able to communicate with the organization easily by telephone, e-mail, and patient portals. This demands careful attention to the accessibility and usability of all messaging channels. For example, clearly posting telephone numbers, streamlining call trees, and usability testing portal interfaces. The Toolkit also emphasizes providing written and audiovisual materials in the patient’s preferred language (including sign language) and implementing multilingual patient portals. Finally, the Toolkit provides resources and guidance to promote cultural sensitivity.

Several recommendations can be improved or expanded to improve DHL. While the Toolkit recommends addressing language differences with audiovisual and written materials (i.e., Tool #9: Address Language Differences), we recommend this strategy for all communication (i.e., Tool #4: Communicate Clearly). Patients often retain less than half of spoken discharge information, with retention varying based on illness severity, cognitive function, and health literacy.^31^ This highlights the importance of supplementing verbal communication with written materials or resources (e.g., digital reminders, follow-up calls, and wrap-around services). We anticipate that innovative transcription software, potentially supported by Artificial Intelligence (AI), will enhance accessibility and comprehension.^32^ Conversational AI systems using large language models will be an increasingly common part of the clinical workflow. However, as with other communication methods, these must be easy to use and provide high-quality information. We also believe synchronous or asynchronous virtual appointments (i.e., telemedicine) offer a scalable and patient-centred strategy to complete brown bag medication reviews. Patients at home or in care facilities can retrieve their medications or recruit a caregiver to assist in providing a history. We have incorporated this strategy into our telemedicine curriculum for medical students and residents.^33^ Further, there are digital tools specifically designed to support medication reconciliation,^20,21^ which may warrant consideration depending on the organizational context. We routinely engage “virtual” interpreters using an internet-enabled tablet computer in our practice. We anticipate that similar services will be progressively easier to integrate into virtual encounters. Finally, resources specific to the Canadian context, such as those related to cultural sensitivity, should be consulted (e.g., Culturally Safe Engagement: What Matters to Indigenous (First Nations, Métis and Inuit) Patient Partners Companion Guide).^34^

### Tools to improve written communication

The Toolkit currently includes language addressing digital forms of written communication. For example, Assess, Select, and Create Easy-to-Understand Materials: Tool #11 directs readers to the US Department of Health and Human Services’ *Health Literacy Online: A Guide for Simplifying the User Experience*.^22^ This evidence-based publication aims to “develop intuitive health web sites and digital tools that can be easily accessed and understood by all users” including people who “struggle to find, process, and use online health information.”^22^ There are five categories of strategies in *Health Literacy Online*^22^:

(1) What we know about users with limited literacy skills,
(2) Write actionable content,
(3) Display content clearly on the page,
(4) Organize content and simplify navigation,
(5) Engage users, and
(6) Test your site with users with limited literacy skills.

The Toolkit cautions against directing patients to web sites without assessing the resources, evaluating patient literacy levels, or providing navigation services (i.e., Use Health Education Material Effectively: Tool #12). Patients must have the skills to use materials (e.g., videos and the internet) and resources to navigate virtual care.^25^ The tool recommends training “patients to use the patient portal and to be discerning consumers of internet content.” This is best achieved whilst observing patients using the portal and collecting feedback using the Patient Portal Feedback Form.^26^ Most questions in the Patient Portal Feedback Form^26^ are binary (yes or no) and encourage open-ended feedback to inform iterative improvement efforts.^26^ Another strong practice includes providing health navigators to assist patients in completing forms, using patient portals, and seeking virtual care.

While the Toolkit includes recommendations for patient-facing technologies, these recommendations require greater specificity. For example, *Health Literacy Online*^22^ offers recommendations specific to mobile devices (e.g., limit typing, use large buttons or tappable areas, and place frequently used buttons in the centre or bottom of the screen).^23^ *Health Literacy Online* also includes a Strategies Checklist^24^ to assess the quality and consistency of implemented strategies. The checklist has been used to make systems (e.g., mobile apps^35^ and personal health records^36^) easier to use and the content easier to understand.

The checklist does not identify all potential improvement opportunities. However, it is easy to use within our digital healthcare ecosystem and can be supplemented with other tools and resources.^37,38^ For example, the Sydney Health Literacy Lab Health Literacy Editor (SHeLL) (available here: https://shell.techlab.works/) is a web-based editor that uses health literacy guidelines to make materials easy to read.^39^

Toolkit guidance to support patients using portals and virtual care is well considered. However, we recommend creating similar tools for other technologies (e.g., remote patient monitoring equipment, apps, and smart devices). The recent uptake of Generative Artificial Intelligence (genAI) will necessitate educating patients about the potential, limitations, and risks associated with genAI tools for improved health literacy. For example, Marasovic and colleagues identified health literacy advantages (e.g., generating and revising materials to improve readability and comprehensibility) and drawbacks (e.g., inaccurate information, introducing bias, and inadequate or inaccurate attribution to sources) using ChatGPT.^40^ Digital maps and wayfinding technology available on self-service kiosks or mobile devices can help patients navigate the built healthcare environment (e.g., hospitals and clinics) and the clinical workflow (e.g., speciality consultations, diagnostics, and other referral services). We recommend ensuring that an organization’s web site includes all the information the Toolkit recommends including it the organization’s brochure (e.g., contact information, services provided, address, and map). Digicasters may be used to showcase information in waiting rooms or other spaces. Patients can use kiosks or tablets to check in for appointments and provide intake information. These nonverbal cues may create more privacy for patients reporting sensitive symptoms.

### Tools to improve self-management and empowerment

The Toolkit contains recommendations for digital support to promote patient self-management and empowerment. For example, it describes how the Question Builder^27^ software enables patients to generate questions in advance of medical appointments to guide discussions and person-centred decision making.^28^ The Toolkit includes best practices for medication reconciliation, adherence, and safety.^28^ It recommends incorporating standardized, plain-language instructions for patients into electronic medication orders.^28^ For those practices that lack the needed technology, the Toolkit includes forms to complete these patient-centred detailed medication lists (e.g., what the medication is for, instructions for taking it, and what times of day it should be taken). The Toolkit also recommends assisting patients when choosing and configuring electronic reminders, apps, smart bottles, and pill boxes.

The Toolkit includes questions health professionals can use with patients to gather feedback using patient portals and secure messaging.^29^ We recommend adding questions to assess other direct-to-consumer devices, apps, or tools. Importantly, the Toolkit cautions against gathering data from patients using exclusively digital tools to avoid excluding specific populations or inadvertently promoting social biases. Finally, the Toolkit encourages including audio options to increase accessibility.

We recommend adding technology features that pose questions directly through portals or secure messaging to inquire about patients’ health status, healthcare goals, recent encounters, and consumer experience. This obviates the need to survey patients at the point-of-care, allowing time for patient reflection, provider discussion, and care planning. Conversational AI systems using large language models can support this task or help patients find current and evidence-based answers to their questions. For example, the World Health Organization created Smart AI Resource Assistant for Health (S.A.R.A.H),^41^ a generative Artificial Intelligence (AI) solution to support health promotion.^42^ S.A.R.A.H. is available in eight languages and covers healthy eating, stress management, and smoking cessation.^41^ In addition to the EHR, other, potentially more comprehensive medication lists may be available and worth consulting or incorporating. Ideally, these medication lists would allow the inclusion of more patient-centred medication regimen details as well. As devices become more affordable and accessible, we recommend leveraging technology to support interventions and/or behaviour change, such as automated pill boxes, digital reminders, and wearables (e.g., activity trackers and fall monitors). As with the deployment of patient portals, these initiatives will need to be monitored to assess the consistency and quality of use and inform iterative product improvement cycles.

### Tools to improve supportive systems for health-related social needs

The Toolkit includes technology recommendations to identify and address social and structural determinants of health. Professionals may use off-the-shelf or bespoke social screening questions embedded in the EHR to document, code, and track health-related social needs. This can streamline referrals to federal, provincial, territorial, or community-based resources. In the United States, for example, there is a health-related social needs questionnaire that can be securely sent to patients’ smartphones when presenting at hospitals, outpatient settings, and other care settings (e.g., pharmacies, mobile clinics, and shelters).^43^ If a social need is identified, the algorithm sends numbers, locations, and links to community resources within the patient’s zip code.^43^ We could envision similar initiatives being successful in Canada in providing recommendations for supports tailored to identified need and geographic location. While the Toolkit also recommends encouraging patients to bring a support person to appointments, we believe technology can augment a standardized workflow to identify and facilitate the inclusion of friends and family during appointment booking.

As an extension, we have context-sensitive technology recommendations for addressing health-related social needs exacerbating the gaps in access to care. Canadian substitutions are needed for Tool 18 (i.e., Attend to Social Needs) and provincial and territorial information must be added to Tool 19 (i.e., Help Patients Pay Less for Medicine). For example, some medications can be subsidized in British Columbia if Special Authority Forms are submitted.^44^ In addition to recommending resources for patients for literacy and math, it is essential to furnish patients with digital literacy supports. Canada Health Infoway’s Digital Health Learning Program offers educational resources for patients to (1) learn about virtual care, (2) become familiar with health data, and (3) explore proactive management behaviours.^45^ Connected Canadians offers free technology training and support for older adults.^30^ With respect to referrals, they the status of referrals could be communicated digitally and electronically send preparation materials (e.g., patient portal and secure messaging). Although it is emphasized that providers need private time with their just their patients if support people attend appointments in person, this needs to be reiterated for virtual care.

## Advancements: Health literacy universal precautions in virtual care

There have been significant advancements in virtual care that foster OHL. Coleman and colleagues published a series of articles describing how universal communication precautions can optimize virtual care.^6,7,46,47^ Key tenets for virtual care include agenda-setting, limiting technical and medical jargon, avoiding information overload, using multi-modal communication, and summarizing discussions, using techniques such as the Teach-Back method. Modified for video and telephonic encounters, these strategies can mitigate misunderstandings, engender patient trust, promote self-efficacy, and reduce healthcare disparities.

Similarly, the Clinician Change Virtual Care Toolkit authored by Canada Health Infoway and Healthcare Excellence Canada is “a general guide to support clinicians with implementation and use of virtual care.”^48^ It offers a checklist to assess patient and caregiver’s needs and preferences, which, in turn, can inform the appropriateness of virtual care. The Toolkit has a table comparing different virtual care modalities (i.e., video, telephone, secure messaging, and remote patient monitoring). It also has a section on health equity, digital health equity, and associated challenges and mitigation strategies.^48^ There are links to patient educational resources,^49^ checklists for virtual appointments, and a list of virtual care offerings within their province or territory.^49,50^

Although these Toolkits and resources offer valuable information for clinicians, they tend to focus disproportionately on *synchronous* videoconferencing (e.g., Clinician Change Virtual Care Toolkit^48^ and Telehealth^51^) over telephone care, secure messaging, and remote patient monitoring. As more information and services are delivered using a variety of technology, developers must invest comparable time and effort into designing usable materials, devices, and software. Thus, we believe it prudent to develop extensible technology-agnostic tools to advance digital health inclusion. Rather than focusing on one type of digital health technology (i.e., virtual care), we propose more generic and flexible recommendations that can be adapted to a wide array of current and emerging technologies.

## Discussion

### Modifications to address the digitalization of healthcare

Our analysis of the Health Literacy Universal Precautions Toolkit identifies significant advances in supporting personalized, context-sensitive, and patient-centred care. However, it also surfaced critical gaps that demand urgent attention. Healthcare organizations must do more than adapt to a rapidly evolving digital environment; they must also assume a leadership role and promote person-centred care when shaping how digital tools are designed, introduced, and integrated into a fragmented and often inequitable ecosystem.

To meet this challenge, our recommendations emphasize four key imperatives. First, we must expand the Organizational Health Literacy (OHL) (e.g., Refs. 5, 10, 52, 53) conceptual framework to include digital health. We have taken the first step by updating the definition of OHL to include digital information and services. This update notwithstanding, an expanded framework must also provide the conceptual scaffolding for new aims, strategies, technologies, implementations, and evaluations. Without this shared foundation, progress will remain piecemeal and challenging to scale.

Second, we must develop new tools to support universal communication precautions within a rapidly evolving digital ecosystem. Building on the foundational work of AHRQ, Canada Health Infoway, and Healthcare Excellence Canada, we envision comprehensive integration of digital modalities into existing health literacy guidance. Coleman’s Universal Communication Precautions for Telehealth is a promising start but requires additions to support the successful implementation of *any* digital health technology, including patient portals, mobile apps, consumer-facing AI, wearables, and remote monitoring systems. Existing Telehealth HHS categories, such as Getting started; Planning your workflow; Preparing patients; and Policy, can provide a starting point to author these updates.

Third, we must account for the sociotechnical and structural determinants shaping digital health experiences. Efforts to modernize health literacy must also address broader contextual challenges, such as healthcare fragmentation, variable technology access, socioeconomic inequality, and international differences in delivery models that threaten to widen healthcare gaps if left unaddressed. Fortunately, there are several current initiatives attempting to narrow the divide through expanding access to free public Wi-Fi^54^ and increasing access to hardware.^55^ Moreover, the Government of Canada is attempting to achieve universal high-speed Internet access by 2030.^56^ Still, more could be done to facilitate people using hardware, technologies, and software for health. For example, partnering with community interest holders (e.g., in schools, libraries, pharmacies, community centres, and places of worship) to not only improve technology and internet access but provide wrap-around services such as written materials, technology support, and care navigation.

Fourth, we must implement targeted, actionable enhancements to the Health Literacy Universal Precautions Toolkit. We offer a detailed gap analysis with recommendations, each aimed at improving the usability, accessibility, and coordination of care through digital platforms. These enhancements foster communication, promote interprofessional collaboration, and elevate the patient experience by centring their needs, goals, comprehension, self-efficacy, and satisfaction. Together, these modifications provide a roadmap to ensure that health literacy efforts remain relevant, equitable, and digitally inclusive in the face of continuous healthcare transformation.

Fifth, given the rapid increase in the number of digital health technology implementations, it is essential to standardize how initiatives are measured and reported to democratize knowledge, track improvements, and compare the factors that contribute to implementation successes and failures. Implementations often face challenges (e.g., inadequate evidence, limited adoption, and funding sustainability) that prevent them from being scaled up beyond pilot studies.^57^ Organizations often fail to disseminate implementation lessons learned^57^ and even when they do (e.g., Leviss^58^) it can be difficult to compare between the projects and draw insights. One potential resource to facilitate structured and standardized reporting is the 20-item Guidelines and Checklist for the Reporting on Digital Health Implementations (iCHECK-DH).^57^ The iCHECK-DH development team have published a wealth of real-world examples to guide similar efforts, improve knowledge sharing, and inventory the facilitators and barriers to success.^57^

### Future work: Tools to improve digital health

This article offers supplemental language and recommendations to enhance tools described in the Health Literacy Universal Precautions Toolkit. These additions are designed to help patients navigate a rapidly evolving digital healthcare ecosystem and structured to align with the trajectory of care delivery as it continues to shift toward digital platforms. These recommendations are also responsive to the emerging omnichannel movement in healthcare service delivery. Driven, in part, by the commercialization of healthcare services and direct-to-consumer marketing, the omnichannel movement seeks to provide a seamless and personalized patient care experience using a network of interconnected modalities, including portals, apps, personal devices, wearables, and virtual services.^59^

While these tech-forward recommendations provide a valuable framework for growth, they do not directly address the widening Digital Divide. The Digital Divide encompasses social factors that limit equitable access to digital care. These include disparities in device ownership, broadband availability, data affordability, digital literacy, and clinician willingness to engage with digital tools. As United Nations Secretary-General António Guterres has warned, digital exclusion is emerging as a new social determinant of health—one that risks compounding existing disparities in care access, outcomes, and experience along lines of race, gender, income, geography, political marginalization, stigma, and disability.^60^ Given these realities, we recommend adding a digital health section that includes tools to support the deployment of digital health equity. Addressing the structural barriers that underpin the digital divide is essential for any health literacy effort that hopes to be inclusive, forward-compatible, and just.

#### A health systems framework to promote digital equity

The digital inverse care law implies that the “people most in need of care are also least likely to have access to, or engage with, technology.” When implementing new technologies, we risk exacerbating the Digital Divide.^61^ Digital health equity is “the provision of equitable health service using digital communication or information tools for the collection, exchange and use of health-related information for purposes of promoting quality care.”^62^

Given the magnitude of the problem, implementing individual technologies without an overarching strategy that accounts for the broader sociotechnical landscape will be insufficient. Richardson and colleagues’ Framework for Digital Health Equity,^63^ which details digital determinants of health at four levels (i.e., individual, interpersonal, community, and societal), is a valuable model for analyzing systems and designing solutions. We believe stakeholders must adopt health systems approaches grounded in three pillars of success: technology, workflow, and advocacy.

We provided a detailed description of technology-focused strategies, including patient assistive tools, usability testing of patient-facing interfaces, and the deployment of standardized, patient-centred language across multiple sensory modalities. We also touched on essential workflow adaptations, such as offering IT support services directly to patients, selecting and tailoring solutions based on patient demographics, attributes, and preferences, and delivering high-touch wrap-around services—including patient navigators, advocates, and family engagement—as adaptive scaffolding for patients adjusting to new models of care.

However, we have not yet addressed in depth the critical role of advocacy, policy, and governance in fostering a digitally inclusive equity ecosystem. Public health leaders and agencies must collect, analyze, and operationalize data on social determinants of health, health literacy, and digital health literacy to optimize service delivery. Underserved communities—both rural populations and urban residents facing the consequences of digital redlining—require affordable, high-speed broadband internet. Healthcare organizations must partner with trusted community institutions such as libraries, schools, community centres, and places of worship to expand access, offer social support, and deliver culturally competent educational programs.

#### Meet patients where they are: give options and alternatives

Helping begins with meeting people where they are.^64^ An individual’s DHL is a constellation of attributes across a continuum, rather than a static single construct. A patient may boast advanced skills with a device, operating system, app, or health topic, but struggle in other areas. Co-designing interventions with patients can illuminate biased and harmful assumptions about their capabilities and move systems towards personalized rather than one-size-fits-all solutions.^65^ For example, offering to print resources for patients, using close-captioning for online encounters, and providing multiple communication channels to contact clinicians or book appointments.

#### A human-centred approach procurement, development, and evaluation

To “help patients to navigate, understand, and use information and services,”^66^ we recommend adopting a Human-Centred Design (HCD) approach.

Human-centred design is an approach to interactive systems development that aims to make systems usable and useful by focusing on the users, their needs and requirements, and by applying human factors/ergonomics, and usability knowledge and techniques. This approach enhances effectiveness and efficiency, improves human well-being, user satisfaction, accessibility and sustainability; and counteracts possible adverse effects of use on human health, safety and performance.^67^

HCD should not only support the development of new systems but also guide the evaluation and refinement of existing systems. Existing systems can be studied using an array HCD methods (e.g., simulations, cognitive walkthroughs, usability heuristic checklists, questionnaires, and remote user monitoring) to improve the alignment between systems, users, needs, requirements, workflows, and contexts.^68,69^

#### Assess for readiness and provide training and support for digital health

Assessing for readiness, and providing training, support, and opportunities for healthcare providers and patients to test digital tools and technologies is imperative to move the digital health transformation forward. For example, the Readiness and Enablement Index for Health Technology (READHY)^70^ can be used to help identify how receptive people might be to digital health tools and technologies and inform tailored supports to increase their receptivity. Additionally, using Healthcare Failure Modes and Effects Analysis^TM^, we have identified a range of technology and workflow problems in telemedicine encounters.^71^ This foundational work informed the design of an eventual telemedicine curriculum for medical and physician assistant students. The curriculum includes a series of simulations to practice telemedicine skills (e.g., troubleshooting technology,^71^ environmental assessment,^72^ and webside manner and safety planning^73^). From the patient perspective, for a telemedicine appointment, an e-mail could include a video tutorial on how to software, allow the user to sign in and test out the software in advance of the appointment, and include phone numbers for telephone support.

## Conclusion

OHL must address the digital transformation of health to better deploy and support digital health technologies for patient information and services. While we have seen advancements made with respect to personal health literacy by introducing terms such as digital health literacy, these need to be paralleled with recognition of the responsibility of organizational as well. In response, we redefined OHL to include digital and analogue information and services to highlight the role of digital health and the changes in demands placed on patients. This definition emphasizes that foundational work in OHL (e.g., equitable access, clear communication, and patient empowerment) is still essential we must augment it with unique considerations are needed to reflect the complexities and demands associated with the deployment of digital health technologies. We took an initial step toward more comprehensively integrating digital health into OHL by identifying where the Health Literacy Universal Precautions Toolkit references digital technologies and proposing opportunities for its expansion. Organizations must proactively identify and mitigate barriers introduced by health technology. This is underscored by the discrepancy observed between the public’s lagging adoption of digital health outpaced by their desire to use it. While others have made progress in this area, most of it has been limited to synchronous virtual care and it is imperative that we have a more generic approach that can guide the support and use of all digital health technologies by patients and increase digital health equity.

We encourage others to continue moving this agenda forward by updating how we conceptualize, operationalize, assess, and support OHL to reflect the changes and increasingly digital nature of healthcare. Organizations must enhance policies, training, and patient engagement strategies that address digital health literacy. New tools need to be developed that address accessibility, privacy, and equity of digital health technologies and include evaluation to ensure they are successful across diverse populations. Moreover, standardized reporting of digital health technology implementations can make it easier to compare between implementations, reveal common causes of success and failure, and therefore improve the effectiveness of these implementations overall. Adopting a holistic understanding of OHL that emphasizes the importance and challenges associated with technical innovations results in organizations that are better positioned to enable all individuals in finding understanding and using health information and services regardless of whether they are analogue or digital. Ultimately, this will result in advancing health equity, improving patient outcomes, narrowing the digital divide, and realizing the potential of digital health.
